# Reliability and validity of a symptom management scale in a sample of Chinese treatment seekers

**DOI:** 10.1192/j.eurpsy.2025.1585

**Published:** 2025-08-26

**Authors:** H. W. Fung, T. Y. N. Tsui, C. H. O. Huang, A. K. C. Chau, S. K. K. Lam, J. Y.-H. Wong

**Affiliations:** 1 School of Nursing, The Hong Kong Polytechnic University, Kowlood, Hong Kong; 2 School of Psychology, Australian Catholic University, Melbourne, Australia; 3 Department of Social Work and Social Administration; 4 Department of Psychiatry, the University of Hong Kong, Pokfulam; 5 Nethersole School of Nursing, The Chinese University of Hong Kong, Shatin; 6 School of Nursing and Health Sciences, Hong Kong Metropolitan University, Kowlood, Hong Kong

## Abstract

**Introduction:**

The Recovery Assessment Scale-Domains and Stages (RAS-DS) is a recently developed measure that builds on the original Recovery Assessment Scale (RAS). Within the RAS-DS, which measures one’s recovery from mental illness, a specific domain is called “Clinical Recovery” (RAS-DS-CR). It extends the “Not Dominated by Symptoms” subscale of the original RAS, which only has three items. The RAS-DS-CR provides a more comprehensive assessment of the sense of control over symptoms and is a promising, easily-administered outcome measure for evaluating early interventions such as psychoeducation and skills training programs.

**Objectives:**

This study examined the psychometric properties of the 7-item RAS-DS-CR in a Chinese sample of treatment seekers.

**Methods:**

We analyzed data from 91 participants from a two-month psychoeducation program (M_age_ = 28.87; SD = 7.84, 89.0% female). At baseline, they exhibited high levels of post-traumatic (mean PCL-5 = 57.18; SD = 14.68) and dissociative (mean DES-T = 47.90; SD = 23.13) symptoms. All participants completed the baseline assessment, 83 completed the 2^nd^ pretest, 58 completed the posttest, and 44 completed the two-month follow-up test. They completed the RAS-DS-CR and other validated self-report measures at each time point.

**Results:**

The RAS-DS-CR revealed good internal consistency (α = .805 to .871) at each time point. Intraclass correlations of two tests taken pre-intervention (ICC = .524, *p* <.001) and post-intervention (ICC = .613, p <.001) indicated moderate test-retest reliability. At each time point, the RAS-DS-CR was significantly correlated with self-esteem (r = .338 to .574), depressive symptoms (r = -.402 to -.486), and PTSD symptoms (r = -.245 to -.462), indicating its construct validity. The paired sample t-test also suggested that participants scored significantly higher on the RAS-DS-CR post-intervention compared to pre-intervention (t = -4.330, *p* < .001; Cohen’s *d* = 0.56), providing evidence for its sensitivity to change.

**Conclusions:**

This study provided new evidence for the reliability and validity of the RAS-DS-CR. The RAS-DS-CR is a short and easy-to-use outcome measure of one’s mental health recovery in terms of the confidence in symptom management.

**Disclosure of Interest:**

None Declared

